# Kinin B_1_ Receptor in Adipocytes Regulates Glucose Tolerance and Predisposition to Obesity

**DOI:** 10.1371/journal.pone.0044782

**Published:** 2012-09-14

**Authors:** Marcelo A. Mori, Vicência Micheline Sales, Fabiana Louise Motta, Raphael Gomes Fonseca, Natalia Alenina, Dioze Guadagnini, Ines Schadock, Elton Dias Silva, Hugo A. M. Torres, Edson Lucas dos Santos, Charlles Heldan Castro, Vânia D’Almeida, Sandra Andreotti, Amanda Baron Campaña, Rogério A. L. Sertié, Mario J. A. Saad, Fabio Bessa Lima, Michael Bader, João Bosco Pesquero

**Affiliations:** 1 Department of Biophysics, Federal University of São Paulo, São Paulo, São Paulo, Brazil; 2 Max-Delbrück-Center for Molecular Medicine (MDC), Berlin, Germany; 3 Department of Internal Medicine, State University of Campinas, Campinas, São Paulo, Brazil; 4 Department of Medicine, Federal University of São Paulo, São Paulo, Brazil; 5 Department of Biosciences, Federal University of São Paulo, São Paulo, Brazil; 6 Department of Physiology, University of São Paulo, São Paulo, São Paulo, Brazil; Instituto Butantan, Brazil

## Abstract

**Background:**

Kinins participate in the pathophysiology of obesity and type 2 diabetes by mechanisms which are not fully understood. Kinin B_1_ receptor knockout mice (B_1_^−/−^) are leaner and exhibit improved insulin sensitivity.

**Methodology/Principal Findings:**

Here we show that kinin B_1_ receptors in adipocytes play a role in controlling whole body insulin action and glucose homeostasis. Adipocytes isolated from mouse white adipose tissue (WAT) constitutively express kinin B_1_ receptors. In these cells, treatment with the B_1_ receptor agonist des-Arg^9^-bradykinin improved insulin signaling, GLUT4 translocation, and glucose uptake. Adipocytes from B_1_^−/−^ mice showed reduced GLUT4 expression and impaired glucose uptake at both basal and insulin-stimulated states. To investigate the consequences of these phenomena to whole body metabolism, we generated mice where the expression of the kinin B_1_ receptor was limited to cells of the adipose tissue (aP2-B_1_/B_1_^−/−^). Similarly to B_1_^−/−^ mice, aP2-B_1_/B_1_^−/−^ mice were leaner than wild type controls. However, exclusive expression of the kinin B_1_ receptor in adipose tissue completely rescued the improved systemic insulin sensitivity phenotype of B_1_^−/−^ mice. Adipose tissue gene expression analysis also revealed that genes involved in insulin signaling were significantly affected by the presence of the kinin B_1_ receptor in adipose tissue. In agreement, GLUT4 expression and glucose uptake were increased in fat tissue of aP2-B_1_/B_1_^−/−^ when compared to B_1_^−/−^ mice. When subjected to high fat diet, aP2-B_1_/B_1_^−/−^ mice gained more weight than B_1_^−/−^ littermates, becoming as obese as the wild types.

**Conclusions/Significance:**

Thus, kinin B_1_ receptor participates in the modulation of insulin action in adipocytes, contributing to systemic insulin sensitivity and predisposition to obesity.

## Introduction

As we enter the 21^st^ century, more than 170 million people worldwide suffer from type 2 diabetes (www.who.int). This disorder is strongly correlated with obesity, being nine among ten type 2 diabetic patients also obese or overweight. The interplay between the pathogenesis of obesity and type 2 diabetes strongly relies on the endocrine functions displayed by the adipose tissue. The white adipose tissue (WAT) secretes molecules in response to metabolic inputs to control key physiological processes of the organism, including glucose homeostasis, lipid metabolism, energy balance, inflammation and vascular homeostasis [1], [2], [3]. Adiponectin, for instance, is an adipocyte-specific hormone that is elevated in the serum of individuals after weight loss to promote insulin sensitivity [4], [5].

Kinins are peptides that participate in a wide range of physiopathological processes. Two G protein-coupled receptors of the rhodopsin family, namely B_1_R and B_2_R, have been shown to bind kinins [6], [7]. While the kinin B_2_R subtype mediates the action of bradykinin (BK), the B_1_R subtype is activated by des-Arg^9^-BK (DBK), a product of the cleavage of BK by carboxypeptidases [6], [7]. B_2_R is ubiquitously expressed, whereas the B_1_R subtype is absent in most tissues during basal conditions but is strongly up-regulated by inflammatory stimuli [6], [7], [8]. Thus, many of the physiological functions described for kinins have been attributed to the activation of the B_2_R, while the B_1_R has been mainly correlated to pathological processes [6], [7], [8].

Initial observations proposing a role for kinins in the regulation of glucose homeostasis date back several decades [9], [10]. These reports showed that BK was produced by the working muscle where it induces glucose uptake. Recent studies confirmed these observations in muscle cells [11] and primary adipocytes [12], showing that stimulation with BK was able to potentiate the insulin effects on promoting glucose uptake, by inhibiting JNK activation [13]. In agreement, B_2_R knockout mice (B_2_^−/−^) exhibited insulin resistance and glucose intolerance [14].

The observed effects of kinins on glucose homeostasis are intuitively associated with the activation of the kinin B_2_R, since this receptor is ubiquitously expressed. However, a growing body of evidence supports the participation of B_1_R in the etiology of diabetes. In 1999, Zuccollo et al. [15] reported for the first time that treatment of mice with the kinin B_1_R specific antagonist [Leu^8^]-DBK could prevent hyperglycemia, insulitis and renal damage induced by low doses of streptozotocin. More recently, our group showed that the kinin B_1_R participates in the regulation of blood glucose levels by promoting the release of insulin by pancreatic β-cells [16]. Furthermore, mice lacking B_1_R (B_1_^−/−^) exhibited improved systemic insulin sensitivity [16] and showed resistance against high fat diet (HFD)-induced obesity [17]. Treatment with a stable selective B_1_R antagonist also prevented rodents from gaining weight on a HFD [17] or on a high glucose diet [18]. This antagonist was also able to increase whole body insulin sensitivity and reverse plasma fatty acids composition changes in a rat model of insulin resistance [18].

Despite the body of evidence that supports a role for the kinin B_1_R in obesity and insulin resistance, the mechanisms through which the B_1_R participates in the pathogenesis of these diseases remain unknown. Our group demonstrate that leptin, a cytokine secreted exclusively by adipocytes, participates in this process [17]. Others showed that the B_1_R blockade may protect from obesity and insulin resistance through inhibition of inammation in adipose tissue. Both hypotheses, which are not exclusive, allude to a potential role for B_1_R in adipose tissue.

In the present study we show that stimulation of constitutively expressed kinin B_1_R in mouse epididymal adipocytes promotes glucose uptake by these cells. Accordingly, adipocytes from B_1_^−/−^ mice exhibit reduced activation of key mediators of the insulin signaling pathway and display decreased glucose uptake. Interestingly, by rescuing the expression of the kinin B_1_R exclusively in cells of the adipose tissue, we partially rescue these phenotypes, as well as the increased systemic insulin sensitivity and the resistance against HFD-induced obesity of B_1_^−/−^ mice. Taken together, our results point to an important role for kinin B_1_R in adipocytes to modulate local and systemic insulin action and predisposition to metabolic diseases.

## Results

### Expression and Functionality of the Kinin B_1_R in Mouse Adipocytes

**Figure 1:**
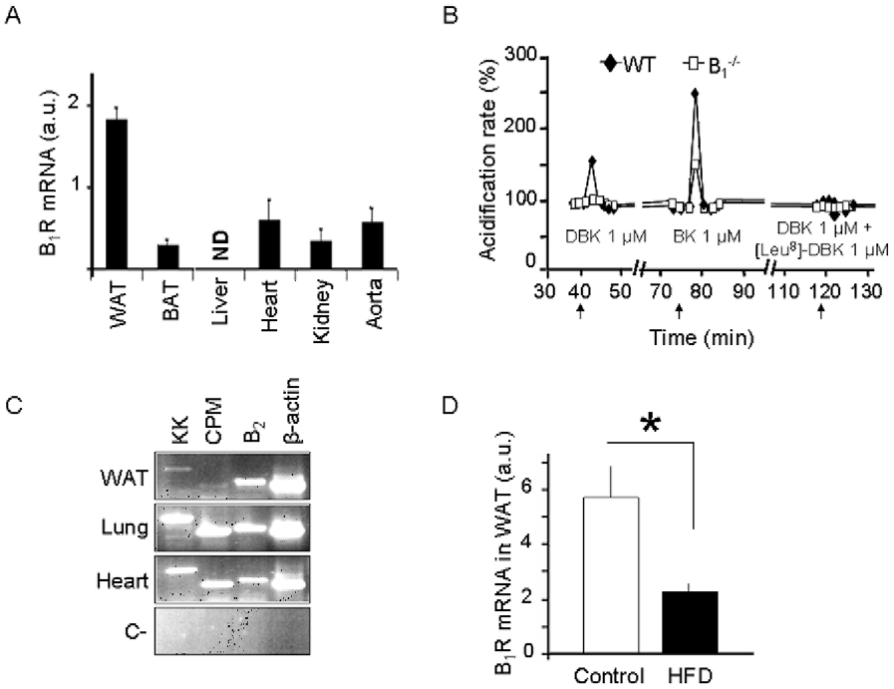
The kinin B_1_R is constitutively expressed in WAT and downregulated with obesity. A: Kinin B_1_R mRNA expression was quantified in different organs of male C57Bl/6 mice (*n* = 5) using TaqMan real-time PCR. Data are expressed as mean±SEM of the 2^−ΔCt^ parameter, which represents the relative expression of B_1_R mRNA in relation to β-actin mRNA. WAT, white adipose tissue; ND, non-detectable; a.u., arbitrary units. B: Effects of acute treatment with the B_1_R agonist des-Arg^9^-bradykinin (DBK), the B_2_ receptor agonist bradykinin (BK) or DBK in the presence of the B_1_R antagonist [Leu^8^]-DBK on the extracellular acidification rate of isolated adipocytes from wild type (WT) and B_1_ knockout (B_1_^−/−^) mice (*n* = 5 animals per group) measured using the *Cytosensor* system. C: Representative RT-PCR showing expression of components of the kallikrein kinin system kallikrein in WAT, lung, and heart of C57Bl/6 male (*n* = 3). KK, tissue kallikrein; CPM, carboxypeptidase M; B_2_, kinin B_2_ receptor. C-, negative control. D: C57BL/6 mice were submitted to HFD for 9 weeks (*n* = 5 per group). Kinin B_1_R expression was quantified in WAT by real time PCR. Results are mean ± SEM. *, *P*<0.05.

To assess basal expression of kinin B_1_R in fat versus other tissues, we performed real-time PCR analysis using total RNA from different organs of adult male C57Bl/6 mice. As shown in Figure 1A, epididymal WAT had the highest basal level of B_1_R mRNA expression among all tissues analyzed. Stimulation of B_1_ and B_2_ receptors with DBK and BK acutely increased acidification rate of the medium in response to the agonists, demonstrating the functionality of both receptors in isolated adipocytes (Figure 1B). In addition, B_1_^−/−^ cells, as well as WT cells treated with the B_1_ antagonist [Leu^8^]-DBK, did not respond to DBK induction, confirming the specificity of the method to detect functional kinin B_1_R. Expression of other components of the kallikrein-kinin system could also be detected in mouse WAT, including B_2_R, carboxypeptidase M and tissue kallikrein (Figure 1C), which along with the previously reported presence of kinin-degrading enzyme ACE [19], indicates that this system is potentially active in the adipose tissue. In addition, B_1_R mRNA was downregulated in epididymal adipose tissue, as well as in heart of obese versus lean mice (Figure 1D and Figure S1). Together, these data demonstrate that the kinin B_1_R is constitutively expressed in fat of mice, suggesting a potential participation of this receptor in adipose tissue function.

### Kinin B_1_R Activation Improves Insulin Sensitivity in Mouse Adipocytes

**Figure 2:**
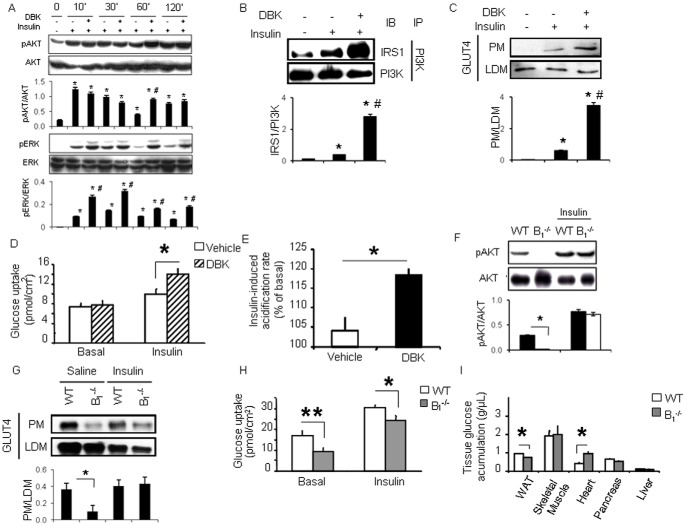
Kinin B_1_R modulates insulin action in mouse adipocytes. A: AKT and ERK phosphorylation upon stimulation of differentiating 3T3-L1 preadipocytes with 10 µg/mL insulin (in the presence of 1 µM dexamethasone, 0.5 mM 3-isobutyl-1-methylxanthine and 10% FBS). *, *P*<0.05; *vs.* non-stimulated; ^#^, *P*<0.05: *vs.* insulin only. The blots and quantification are one representative experiment out of three performed independently. B: 3T3-L1 adipocytes were incubated with or without 1 µM DBK for 90 minutes prior to 10 min stimulation with 1 µg/mL insulin. Cells were harvested for immunoprecipitation (IP) followed by immunoblotting (IB) or C: membrane fractionation for assessment of GLUT4 translocation by western blotting. *, *P*<0.05; *vs.* non-stimulated; ^#^, *P*<0.05: *vs.* insulin only. The blots and quantification are one representative experiment out of two performed independently. D: Isolated epididymal adipocytes of WT mice were incubated with 5 mM glucose media with or without 1 µM DBK for 90 minutes and [^3^H]-2-deoxy-glucose accumulation was measured under basal conditions or after 15 min stimulation with 10 nM insulin. Data represent mean ± SEM of at least 8 independent samples obtained from an adipocyte pool of 15 animals. *, *P*<0.05 *vs.* respective control. E: Effect of 90 minutes incubation with 1 µM DBK on the insulin-induced extracellular acidification rate of 3T3-L1 adipocytes measured by *Cytosensor* system (*, *P*<0.05; *n* = 4 per group). Data represent mean ± SEM of extracellular acidification. F: Random fed WT or B_1_^−/−^ mice (*n* = 4 per group) were injected in the vena cava with either saline or a 10 U insulin bolus. Epididymal fat was harvested after 10 minutes for assessment of AKT phosphorylation by western blotting or G: GLUT4 translocation. H: Glucose uptake was assessed in isolated epididymal adipocytes of WT and B_1_^−/−^ cells under basal conditions or after 15 min stimulation with 10 nM insulin. Data represent mean ± SEM of at least 8 independent samples obtained from an adipocyte pool of 15 animals. *, *P*<0.05; **, *P*<0.001 *vs.* respective control. I: Fasted mice received 0.2 U human insulin per kg, 0.083 mCi [^3^H]-2-deoxy-glucose per kg was subcutaneously injected, mice were sacrificed and tissue collected. Data show mean ± SEM of 5 animals per genotype. *, *P*<0.05.

BK has been shown to promote insulin sensitivity in adipocytes via activation of the kinin B_2_R [12], [13], [20]. To test the hypothesis that the agonist of the B_1_R subtype could also mediate similar effects on fat cells, we assessed insulin signaling in differentiating 3T3-L1 adipocytes in the presence or absence of DBK. AKT phosphorylation, an event that occurs within 10 minutes upon insulin stimulation, was not potentiated by DBK, however dephosphorylation of AKT, which occurs later during the time-course, was abrogated in cells pre-incubated with the B_1_R agonist (Figure 2A). Furthermore, insulin-induced ERK phosphorylation was increased by over 2-fold with DBK treatment (Figure 2A). Consistent with this, pre-incubation of 3T3-L1 adipocytes with DBK for 90 minutes significantly increased insulin-induced association of the p85 regulatory subunit of PI3K to IRS1, an event that precedes AKT phosphorylation (Figure 2B). In addition, DBK was able to potentiate insulin-induced GLUT4 translocation to the plasma membrane in 3T3-L1 adipocytes (Figure 2C) and insulin-induced glucose uptake by isolated adipocytes (Figure 2D). DBK alone did not affect glucose uptake, suggesting an insulin dependent phenomenon. In agreement, incubation of 3T3-L1 adipocytes with DBK significantly augmented insulin-induced increase in cell acidification rate (4.5 fold, P<0.05), which again demonstrates the positive effect of B_1_R stimulation on insulin action in fat cells (Figure 2E). We further investigated insulin signaling and the regulation of GLUT4 translocation and glucose uptake in adipocytes from B_1_^−/−^ and WT mice. Interestingly, AKT phosphorylation during random fed states was dramatically reduced in B_1_^−/−^ versus WT mice, but no difference was observed between these mice in response to acute insulin stimulation (Figure 2F). Moreover, GLUT4 translocation to the plasma membrane (Figure 2G) and glucose uptake (Figure 2H) were significantly reduced in B_1_^−/−^ isolated adipocytes in comparison to WT, most evidently at basal states, but also during insulin-stimulated states. These results demonstrate that the kinin B_1_R modulates the basal status of activation of the insulin signaling pathway in mouse adipocytes, affecting glucose uptake by these cells.

To investigate whether these effects were restricted to the adipose tissue, we measured accumulation of [^3^H]-2-deoxy-glucose in different insulin responsive organs of B_1_^−/−^ and WT mice (Figure 2I). Consistently, glucose uptake by the WAT of B_1_^−/−^ mice was significantly decreased in comparison to the WAT of WT mice. On the other hand, glucose accumulation by the heart was elevated in B_1_^−/−^ mice when compared to WT animals.

### Generation and Characterization of aP2-B_1_/B_1_^−/−^ Mice

In order to study the importance of kinin B_1_R expression to adipose tissue function and whole body metabolism, we generated mice with exclusive expression of B_1_R in adipose tissue. To direct adipose tissue-specific expression, the coding region of the mouse kinin B_1_R gene was cloned downstream of the adipose-specific aP2 promoter (Figure S2A). This construct was injected into the pro-nuclei of C57BL/6 mouse oocytes and positive founders were screened by PCR (Figure S2B) and Southern Blotting (Figure S2C). At least two transgenic lines were generated, but line 25 was chosen to be crossed to B_1_^−/−^ mice to originate aP2-B_1_/B_1_^−/−^ mice due to high expression levels of the transgene in all fat depots analyzed (Figure S2D). In addition, binding assays demonstrated the presence of kinin B_1_R in the membrane of aP2-B_1_/B_1_^−/−^ isolated adipocytes (Figure S2E). Finally, stimulation of aP2-B_1_/B_1_^−/−^ adipocytes with DBK was able to augment basal cellular metabolism, indicating a functional rescue of kinin B_1_R in the fat cells of aP2-B_1_/B_1_^−/−^ mice (Figure S2F).

### Selective Expression of Kinin B_1_R in Adipocytes Rescues Gene Expression Patterns and Glucose Uptake of B_1_^−/−^ Mice

Ablation of kinin B_1_R had a substantial impact on gene expression in adipose tissue, as revealed by microarrays followed by Gene Set Enrichment Analysis (GSEA) [21] (Figure S3A). To determine how the exclusive expression of the kinin B_1_R in adipose tissue could rescue these patterns, we compared B_1_^−/−^ and aP2-B_1_/B_1_^−/−^. Interestingly, some gene sets found to be differentially expressed between WT and B_1_^−/−^ were also found to be differently expressed between aP2-B_1_/B_1_^−/−^ and B_1_^−/−^, including the “DNA Dependant ATPase Activity” and “Detection of External Stimulus” gene sets (Table S1).

**Figure 3:**
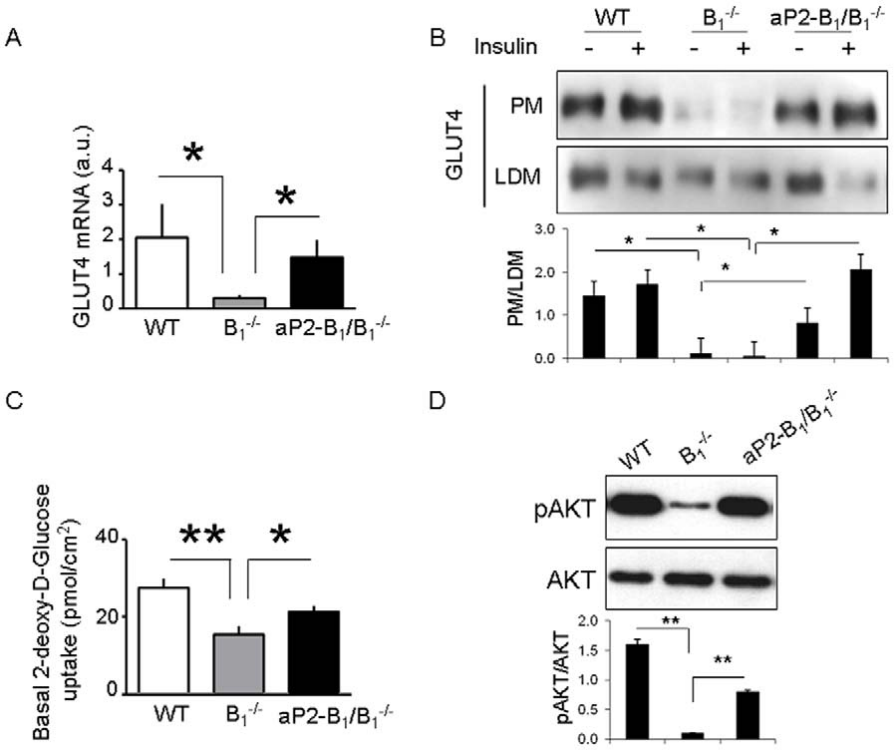
Rescue of kinin B_1_R expression in adipocytes of kinin B_1_R knockout mice (B_1_^−/−^) partially rescues GLUT4 expression and function in adipose tissue. A: Expression of GLUT4 mRNA in epididymal fat of random fed mice measured by realtime PCR. B: GLUT4 translocation in epididymal fat at basal states or after insulin stimulation (10 U intravenous bolus for 10 min). The blots and quantification are one representative experiment out of two performed independently. C: Basal glucose uptake in isolated adipocytes. [^3^H]-2-deoxy-glucose accumulation was measured during 5 minutes. Data represent mean ± SEM of six animals per group. D: Basal AKT phosphorylation in the epididymal fat of random fed mice. The blots and quantification are one representative experiment (pool of 4–5 mice each) out of two performed independently. *, P<0.05; **, P<0.001.

To investigate more specifically the pathways associated with insulin signaling, we generated heat maps to display genes that are known to be regulated by insulin (Figure S3B). In B_1_^−/−^ mice, many of these genes were downregulated in comparison to WT, and part of this pattern was rescued in aP2-B_1_/B_1_^−/−^ mice. To elaborate and validate these observations, we measured the expression of GLUT4 (Figure 3A) and other genes involved in glucose and lipid metabolism (Figure S4) by real-time PCR. Accordingly, the expression of GLUT4 was reduced by 5-fold in B_1_^−/−^ when compared to WT and completely rescued in aP2-B_1_/B_1_^−/−^ mice (Figure 3A). GLUT1, on the other hand, was reduced in both B_1_^−/−^ and aP2-B_1_/B_1_^−/−^ mice in comparison to WT mice (Figure S4). As a surrogate for a functional readout, we also measured GLUT4 translocation in adipose tissue (Figure 3B) and glucose uptake in isolated adipocytes (Figure 3C). The total lack of kinin B_1_R impaired both basal GLUT4 expression in the plasma membrane as well as basal glucose uptake, but the presence of these receptors exclusively in adipocytes was able to rescue these phenotypes. Consistently, the aP2-B_1_ transgene also rescued the decreased basal AKT phosphorylation of B_1_^−/−^ mice (Figure 3D). Together, these results demonstrate that the kinin B_1_R regulates the insulin signaling pathway in adipose tissue.

In certain situations where the kinin B_1_R is absent, the B_2_R is shown to be upregulated [22], which led us to ask whether the expression of this receptor was affected by the ablation of the B_1_R, possibly supporting an effect on insulin signaling. However, despite a trend towards an increase in kinin B_2_R mRNA expression in the aP2-B_1_/B_1_^−/−^ mice versus the WT or the B_1_^−/−^ mice, no significant differences were observed among the groups (Figure S3C), indicating that alterations in B_2_R expression are not sufficient to explain how modulation of the B_1_R affects insulin sensitivity in adipose tissue.

### aP2-B_1_/−B_1_^−/−^ Mice are Lean but Insulin Resistant

**Figure 4:**
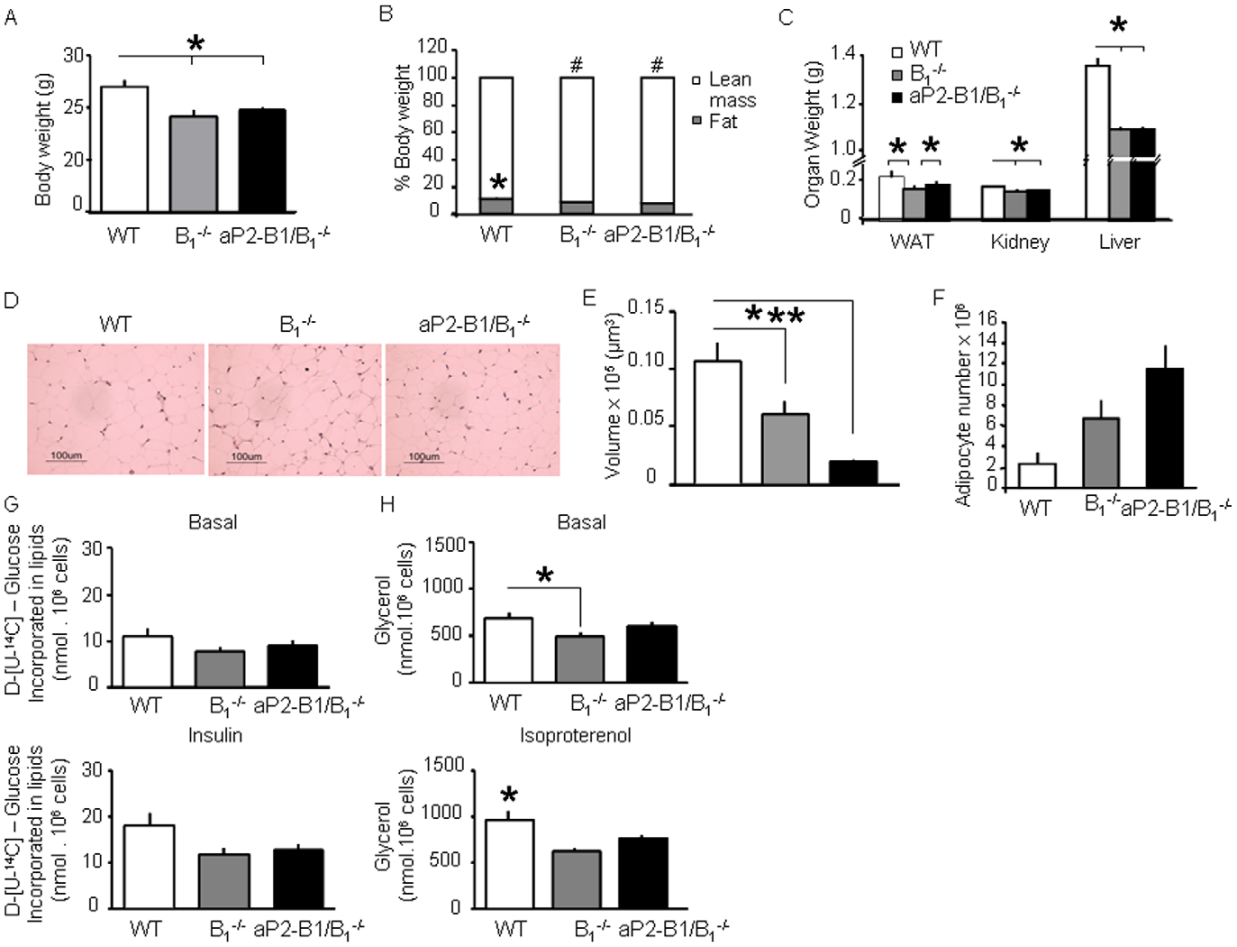
aP2-B_1_/−B_1_^−/−^ mice are lean. A: Body weight. B: Body composition. *, *P*<0.05 vs. B_1_^−/−^ and aP2-B_1_/B_1_^−/−^, fat mass comparison; #, *P*<0.05 vs. WT, lean mass comparison. C: Organ weight. D: Histology of adipose tissue (H&E staining). Representative pictures of at least five animals per group. E: Adipocyte volume. F: Adipocyte number. G: Basal and maximum (insulin-stimulated) lipogenesis in isolated adipocytes. H: Basal and isoproterenol-induced lipolysis as determined by glycerol production by isolated adipocytes. Values are means ± SEM of six animals per group. *, *P*<0.05; **, *P*<0.001.

Next, we wanted to determine how the expression of kinin B_1_R in adipocytes could impact whole body metabolism. Under chow diet, body weight (Figure 4A), food intake, spontaneous activity and body temperature of aP2-B_1_/B_1_^−/−^ mice were similar to B_1_^−/−^ mice (Table S2). However, despite the number of pathways that appeared to be rescued in adipose tissue of aP2-B_1_/B_1_^−/−^ mice, these mice were leaner than the WT (22.3% leaner) and as lean as the B_1_^−/−^ (Figure 4B). Liver and kidney weights were also reduced in B_1_^−/−^ and aP2-B_1_/B_1_^−/−^ mice, suggesting decreased glycogen and/or fat storage in these organs (Figure 4C). However, epididymal fat pad weight was slightly higher in aP2-B_1_/B_1_^−/−^ mice versus B_1_^−/−^ mice (Figure 4C). Surprisingly, morphological analysis of this fat depot revealed smaller adipocytes in aP2-B_1_/B_1_^−/−^ mice in comparison to WT mice or B_1_^−/−^ (Figure 4D and E). On the other hand, the number of adipocytes in the epididymal fat depot of aP2-B_1_/B_1_^−/−^ mice was increased in comparison to the WT (∼6-fold) and to B_1_^−/−^ mice (∼2-fold) (Figure 4F). To assess the rate of lipogenesis in the adipose tissue of these animals, we isolated adipocytes from epididymal fat depots and measured incorporation of glucose into triglycerides (Figure 4G). Basal lipogenesis was not different between groups, but insulin-stimulated lipogenesis was decreased in both aP2-B_1_/B_1_^−/−^ and B_1_^−/−^ mice when compared to WT. When comparing aP2-B_1_/B_1_^−/−^ and B_1_^−/−^ mice to WT mice, although the values did not reach statistical significance, they revealed a strong tendency to be reduced (Figure 4G). Consistently, the expression of the mRNA of lipogenic enzymes such as fatty acid synthase (FAS) and acetyl-CoA carboxylase (ACC) was reduced in aP2-B_1_/B_1_^−/−^ and B_1_^−/−^ mice in comparison to WT mice (Figure S4). Basal and isoproterenol-induced lipolysis was also reduced in B_1_^−/−^ mice (Figure 4H). However, exclusive kinin B_1_R expression in adipocytes could partially rescue this phenotype, since the basal lipolysis in aP2-B_1_/B_1_^−/−^ adipocytes was not significantly different from the WT controls. On the other hand, isoproterenol did not stimulate lipolysis in aP2-B_1_/B_1_^−/−^ as in WT mice and the rate of lipolysis remained similar to B_1_^−/−^ animals (Figure 4H). Furthermore, we investigated the impact of these changes to blood lipid and cholesterol levels of these animals. These modest, but significant alterations (Table S3), namely, lower HDL cholesterol and higher LDL cholesterol levels in B_1_^−/−^ mice in comparison to WT mice were rescued in aP2-B_1_/B_1_^−/−^ mice. These results suggest that the kinin B_1_R in adipocytes might play a role in lipid turnover and participate in the pathogenesis of cardiovascular diseases [23].

**Figure 5:**
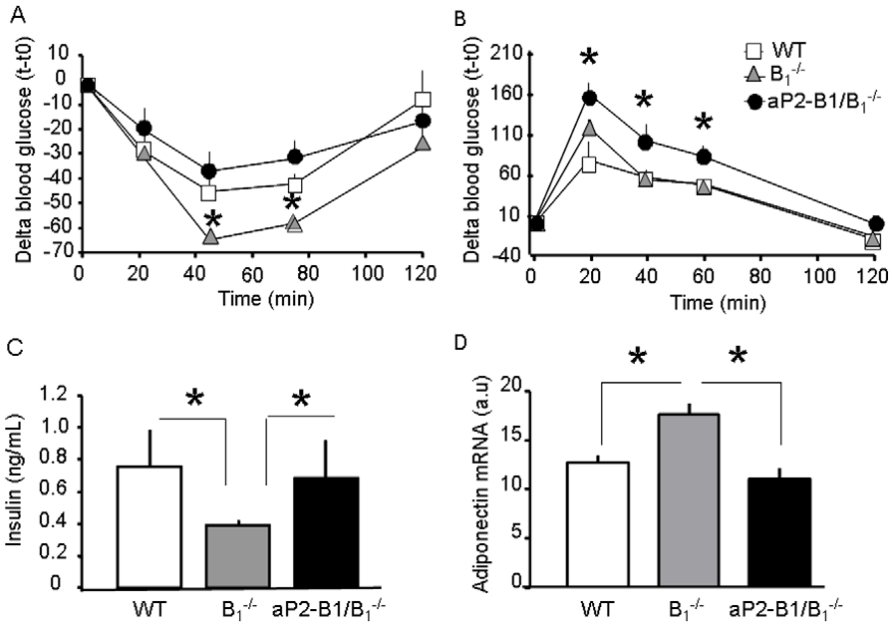
Kinin B_1_R in adipocytes contributes to glucose homeostasis. A: Insulin tolerance test; *, vs. WT and B_1_^−/−^. B: Glucose tolerance test; *, vs. WT and B_1_^−/−^. C: Serum insulin levels. D: Adiponectin expression as quantified by realtime PCR. Values are means ± SEM of six animals per group. *, *P*<0.05.

Reduced fat mass is usually accompanied by an improvement in systemic insulin sensitivity. Thus, despite insulin resistance in adipose tissue, B_1_^−/−^ mice exhibited improved whole body insulin sensitivity and decreased serum insulin levels in comparison with WT mice [16]. Interestingly though, aP2-B_1_/B_1_^−/−^ mice were as insulin sensitive as WT mice despite showing reduced fat mass and smaller adipocytes (Figure 5A and C). However, these mice showed impaired glucose tolerance when compared to the WT controls (Figure 5B). These data inversely correlate with the expression of adiponectin mRNA in adipose tissue (Figure 5D), which has been shown to be a bona-fide marker for whole body insulin sensitivity in mice and humans [4]. Thus, these data suggest that signaling through the kinin B_1_R in adipocytes regulates systemic insulin resistance independently on the adiposity status of the organism. Also, they reinforce the notion that whole body insulin sensitivity correlates inversely with selective adipose tissue insulin sensitivity, a phenomenon that is corroborated by several other studies [24], [25], [26], [27].

### Kinin B_1_R Expression in Adipocytes Controls Predisposition to Obesity

**Figure 6:**
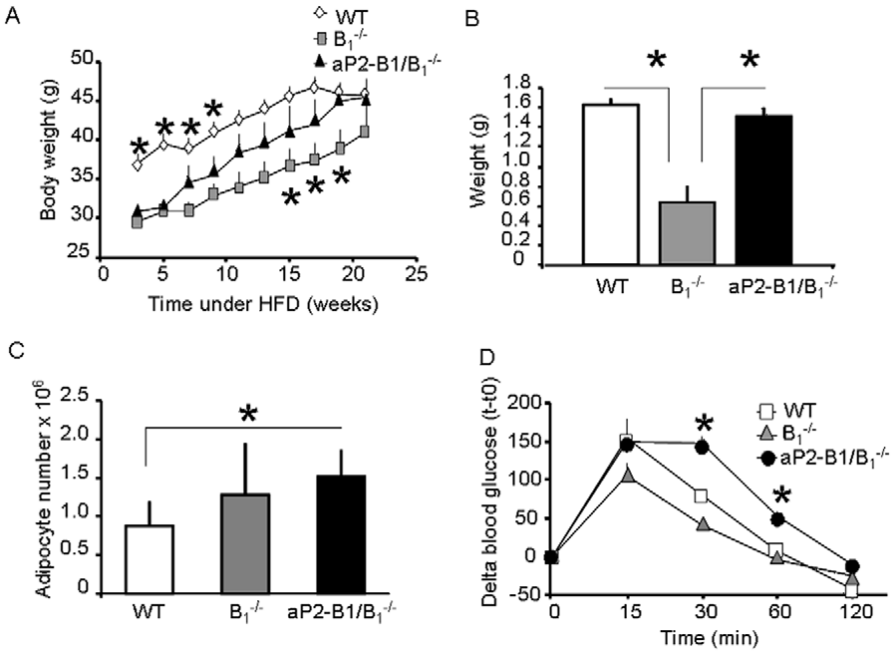
Kinin B_1_R in adipocytes contributes to fat accumulation and insulin resistance in response to a high fat diet (HFD). Animals were submitted to HFD. A: Body weight was monitored during the diet. *, vs. aP2-B_1_/B_1_^−/−^; B: Mice were sacrificed at the end of the treatment and the epididymal fat pads were weighed. C: Adipocyte number was quantified in the epididymal fat pad. D: Glucose tolerance was performed 12 weeks after the beginning of the HFD; *, aP2-B_1_/B_1_^−/−^ vs. B_1_^−/−^ and WT. Values are means ± SEM of six animals per group. *, P<0.05.

B_1_^−/−^ mice are protected against obesity induced by HFD [17] and insulin action in fat has been shown to be required for age-related and hypothalamic lesion-induced obesity [27]. Given the role of B_1_R in regulating insulin signaling in adipocytes, we asked whether its constitutive expression in fat could contribute to the pathogenesis of obesity. We therefore monitored body weight gain and adiposity of aP2-B_1_/B_1_^−/−^, B_1_^−/−^, and WT mice subjected to HFD for 21 weeks. After 7 weeks on HFD, body weights of aP2-B_1_/B_1_^−/−^ and B_1_^−/−^ mice were significantly lower than WT controls (Figure 6A). However, aP2-B_1_/B_1_^−/−^ mice progressively gained weight as mice were maintained on HFD and ended up matching the values of the WT. On the other hand, B_1_^−/−^ mice were significantly lighter than the WT throughout the whole diet (Figure 6A). After 4 months under HFD, mice were sacrificed and epididymal fat was weighed, revealing a complete rescue of adiposity in aP2-B_1_/B_1_^−/−^ mice (Figure 6B). This was linked to atypical hyperplasia of adipocytes in adipose tissue of aP2-B_1_/B_1_^−/−^ mice (Figure 6C), but not adipocyte hypertrophy (Figure S5). Glucose tolerance test also showed that aP2-B_1_/B_1_^−/−^ mice were more glucose intolerant than WT mice after HFD (Figure 6D), even though the level of adiposity was similar between these two groups. These results indicate that the presence of the kinin B_1_R in adipocytes contributes to fat accumulation and insulin resistance in response to HFD.

## Discussion

In most physiological processes associated with the kallikrein-kinin system, the B_1_R is attributed a secondary role, probably due to the fact that this receptor is absent or very low expressed in most mammalian cell types [6], [7]. Our results show that under physiological conditions the kinin B_1_R is functionally expressed in adipose tissue of mice and, similarly to the B_2_R, can promote insulin signaling and glucose uptake in these cells. Our findings do not only define the kinin B_1_R and the bioactive metabolites of BK as important physiological mediators, but also contribute to the understanding of the mechanisms underlying the effects of kinins on the regulation of glucose homeostasis.

Kinin B_1_ and B_2_ receptors interact with G_q_ and G_i_ proteins to activate redundant signaling pathways that include phosphatidylinositol hydrolysis, elevation of intracellular Ca^2+^, arachidonic acid release, eicosanoid production, as well as endothelial nitric oxide synthase (eNOS) activation and nitric oxide production [6], [7]. It is assumed that in situations where the two kinin receptors are co-expressed they may act synergistically to mediate similar cellular processes [6], [7]. However, due to differences in expression pattern and post-activation desensitization between these receptors, the situations when they may act synergistically are normally restricted to pathological conditions when the B_1_R are abundantly expressed. According to our results, this synergism may happen under physiological conditions in adipocytes. Thus, we surmise that the regulation of insulin action in fat cells by kinin receptors are mediated by a congruent mechanism, most likely involving eNOS-mediated inhibition of Jun NH2-terminal kinase [13].

Even though sharing similar functions, kinin receptors may play different roles. This is supported by a body of evidence showing that phenotypes observed in B_1_^−/−^ mice are often antagonistic to those observed in B_2_^−/−^ mice. For example, while B_2_^−/−^ mice exhibit normoglycemia, hyperinsulinemia and systemic insulin resistance [14], B_1_^−/−^ mice are hypoglycemic, hypoinsulinemic and more tolerant to the systemic effects of insulin. Most likely, these differences may be related to differences concerning the expression pattern of these receptors. In this study, we bring further evidence to support this hypothesis. Here we show that the kinin B_1_R is expressed in WAT of mice under normal conditions where it promotes local insulin signaling and increases local glucose uptake. Insulin plays important regulatory roles in the adipose tissue controlling glucose and lipid utilization, adipocyte differentiation and the expression of adipocyte-derived hormones that regulate whole body metabolism [1], [2], [28]. Thus, proper insulin action at the level of the adipose tissue is thought to be required for normal metabolic homeostasis, which include the regulation of whole body insulin sensitivity, the susceptibility to obesity, and the relationship between plasma leptin and body weight [27]. Several animal models corroborate this view and suggest that selective insulin resistance in adipocytes can be otherwise considered an etiological factor leading to improved systemic insulin responsiveness and protection against HFD-induced obesity.

For example, in fat-specific insulin receptor knock-out mice (FIRKO), severe insulin resistance in adipocytes results in leanness and robust protection against obesity and glucose intolerance [27]. Also, specific ectopic expression of a transmembrane isoform of TNFα in adipocytes is able to decrease whole body adipose mass and induce local insulin resistance, while promoting systemic insulin sensitivity [26]. Overexpression of the suppressor of cytokine signaling 3 in adipocytes equally results in local insulin resistance despite increasing whole body glucose tolerance [25]. Moreover, glutamine supplementation, which was shown to specifically induce insulin resistance in the adipose tissue of rats under HFD, also leads to amelioration of insulin action in other insulin responsive organs [24].

Here we show that kinin B_1_R in adipocytes contribute to the regulation of systemic insulin sensitivity and predisposition to HFD-induced obesity. By rescuing the expression of these receptors in adipocytes of B_1_^−/−^ mice, basal insulin signaling and glucose uptake are rescued in these cells. As a consequence, whole body insulin sensitivity of aP2-B_1_/B_1_^−/−^ is normalized to the WT levels, despite lower adiposity. Due to adipocyte-specific kinin B_1_R expression, aP2-B_1_/B_1_^−/−^ mice also gain more weight than B_1_^−/−^ mice on a HFD.

The adipose tissue relies on the production of adipokines to mediate the endocrine functions that regulate whole body metabolism. Adipokines are strongly regulated by insulin and by the metabolic status of the adipocytes [1], [2], [3], [28]. For example, adipose tissue insulin resistance in rats fed with a glutamine-supplemented diet is accompanied by reduced TNFα and interleukin-6 levels, as well as augmented adiponectin levels, which seem to be the cause of increased insulin sensitivity in other organs [24]. Adiponectin is an anti-diabetic hormone produced exclusively by adipocytes which improves glucose utilization and reverses insulin resistance associated with obesity [4]. Decreased expression of adiponectin correlates with insulin resistance in mouse models of altered insulin sensitivity [4], [5]. In B_1_^−/−^ mice, adiponectin expression is increased and may therefore contribute to the improved systemic insulin sensitivity of these mice. However, in aP2-B_1_/B_1_^−/−^ mice, despite being as lean as B_1_^−/−^ mice, adiponectin expression levels are similar to the WT.

In a recent report, Dias and Couture [18] demonstrated that pharmacological blockade of the B_1_R protected rats against obesity and insulin resistance, and that these phenotypes were associated with decreased inflammation in retroperitoneal adipose tissue. Given the close link between insulin resistance and inflammation in adipose tissue, one might expect, based on these data, that B_1_^−/−^ mice would exhibit local insulin sensitivity, whereas we have observed the opposite phenotype. This apparent discrepancy might be related to species or depot heterogeneity, since Dias and Couture used retroperitoneal adipose tissue of rats while we use epididymal adipose tissue of mice. These differences are rather significant to the phenotype, since the B_1_R is absent in retroperitoneal adipose tissue of rats but is constitutively present in epididymal adipose tissue of mice. Nevertheless, both studies agree with the importance of the B_1_R in controlling adipose tissue function and regulating whole body susceptibility to insulin resistance and obesity.

In summary, we demonstrate that kinin B_1_R are constitutively expressed in adipocytes and control insulin signaling, glucose uptake, FFA synthesis (via lipolysis) and adiponectin expression in these cells. The roles of B_1_R in adipocytes have systemic contributions to metabolic homeostasis, since exclusive expression of these receptors in fat cells is able to control whole body insulin sensitivity and predisposition to obesity. These data highlight the importance of kinins in the pathogenesis of diabetes and bring forth novel and more specific targets for clinical interventions.

## Materials and Methods

### Animals

B_1_^−/−^ mice [29] were backcrossed for 10 generations with C57Bl/6 mice (Taconic, Germantown, NY). Animals were obtained from the Federal University of São Paulo, Brazil and from the Max-Delbrück-Center for Molecular Medicine, Berlin-Buch, Germany. All experiments reported were conducted as stated in the National Institutes of Health guide for the care and use of laboratory animals (Institute of Laboratory Animal Resources, National Academy Press, Washington DC, 1996) and approved by a local animal care and use committee. Animals were maintained on standard mouse chow diet at 22°C on 12 h light-dark cycle allowed *ad libitum* access to food and tap water. Adult males were used in all experiments. Food consumption and body weight were monitored weekly in single-caged animals.

### Cell Culture

3T3-L1 cells were grown in culture dishes containing DMEM (Invitrogen) with 25 mM glucose and 10% fetal bovine serum (FBS) (Invitrogen) at 37°C under a 5% CO_2_ atmosphere. For adipocyte differentiation, cells were maintained at total confluence for two days before switching the medium to DMEM with 25 mM glucose containing 10% FBS, 10 µg/mL insulin, 1 µM dexamethasone, and 0.5 mM 3-isobutyl-1-methylxanthine (Sigma-Aldrich). After two days, differentiation medium was replaced by DMEM with 25 mM glucose containing 10% FBS and 10 µg/ml of insulin, and exchanged every second day. After four days, differentiated adipocytes were used for subsequent experiments.

### Microphysiometer Analysis

Adipocytes were isolated from epididymal fat pads [30] and 5×10^5^ isolated adipocytes were fixed in agarose matrix (Molecular Devices). The alterations in pH of the medium as a consequence of basal cellular metabolism was measured every 2 minutes using the Cytosensor microphysiometer [31] (Molecular Devices). Under continuous pH monitoring, we exposed cells acutely (10 s) to 1 µM DBK or BK (Bachem) and measured the variation of acidification rate of the medium, which reflected the pharmacological response of the cells to the agonist. 1×10^6^ 3T3-L1 adipocytes were also coupled to the microphysiometer and stimulated with 10 nM of insulin prior to and after incubation with 1 µM DBK. We quantified the differences in insulin-induced alterations of basal metabolism of cells after chronic stimulation of the kinin B_1_R.

### Glucose Uptake

In *ex vivo* experiments, isolated adipocytes were incubated for 1 hour in the presence or absence of 10 nM insulin. Basal and insulin-stimulated rates of [^3^H]-2-deoxy-glucose accumulation were evaluated [32]. In order to assess *in vivo* [^3^H]-2-deoxy-glucose accumulation by different organs, mice were fasted for 12 hours prior to the beginning of the experimental procedures. Conscious animals were given a subcutaneous injection of 0.083 mCi [^3^H]-2-deoxy-glucose per kg body weight followed after 10 minutes by an intraperitoneal injection of 0.2 U human recombinant insulin per kg body weight. One hour after the first injection, mice were sacrificed by decapitation for collection of blood and organs. After weighing, organs were homogenized in scintillation medium and β-emission was analyzed using a Packard β-counter. β-counts in organ homogenates were normalized by organ weight and then divided by the β-counts in serum.

### Lipolysis and Lipogenesis Assays

Assays in isolated adipocytes were performed as described [33]. As a surrogate for lipogenesis, we measured the incorporation of D-[U-^14^C]-glucose into lipids at basal and in response to insulin (10 nmol/L). Lipolysis was estimated by the quantification of glycerol released in the incubation medium by isolated adipocytes at basal and isoproterenol (10^−5^ M) stimulated conditions.

### Binding Assays

Isolated adipocytes were transferred to 6-well culture plates (2×10^6^ cells/well) 24 h prior to the beginning of the assay. Experiments were performed at 4°C and initiated by the addition of 50 pM [^3^H]-Des–Arg^10^–kallidin in the presence of various amounts of unlabeled peptides as competitors. The binding buffer consisted of 25 mM Tris–HCl, pH 7.4, including 5 mM MgCl_2_, 0.1% bovine serum albumin, and 100 µg/ml bacitracin (Sigma, St. Louis, MO, USA). All measurements were performed in duplicate. The competition binding profiles were analyzed by nonlinear regression analysis using PRISM 5 (Graph-Pad5 Software) [34].

### Gene and Protein Expression

RT-PCR and Western blotting were performed as described elsewhere [17].

### Membrane Fractioning

Cells and tissues were homogenized in cold buffer A (250 mM sucrose; 10 mM tris pH 7.4; 0.5 mM EDTA; Complete protease inhibitors), homogenates were centrifuged at 16,000×g for 20 min, pellets were resuspended in the same buffer and layered on top of buffer B (1.12 M sucrose; 10 mM tris pH 7.4; 0.5 mM EDTA). Samples were centrifuged at 158,000×g for 20 min, the interface was removed with a syringe, diluted in buffer A, and centrifuged at 74,000×g for 9 min. The pellet from this centrifugation, containing the plasma membrane fraction, was resuspended in TNET buffer (1% triton X-100; 150 mM NaCl; 20 mM tris pH 8.0; 2 mM EDTA). The supernatant from the initial centrifugation was recentrifuged at 43,000×g for 30 min. The resultant supernatant was centrifuged at 39,000×g for 75 min and the pellet, containing the low density microsomes fraction, was resuspended in TNET buffer [35].

### RNase Protection Assay and Southern Blotting

Expression of the aP2-B_1_ transgene was determined using RNase protection assay (RPA III kit, Ambion). The probe corresponded to the antisense transcript resulted of the amplification of the transgene using the following primers: 5′-TGGACTTCAGAGGCTCATAG-3′ and 5′-CCAGCAACCTGTAGCGGTCC-3′. The PCR fragment was cloned into pGEM-T Easy vector and used as template for *in vitro* transcription. For Southern Blotting, the same PCR fragment was purified through a silica gel column and labeled with α-[^32^P]-CTP using the Prime-It kit Random Primer Labeling RmT (Ambion). Genomic DNA was digested with Sac I and subjected to 1% agarose gel electrophoresis. DNA was depurinized, neutralized and then transferred by capillarity to a Hybond-N nylon membrane (GE Healthcare) and cross-linked using UV. The membrane was incubated overnight at 62°C with 50 mM Tris pH 8.0, 10 mM EDTA, 5×SSC, 1×Denhardt’s, 0.2% SDS, 100 mg/mL salmon sperm DNA containing the labeled probe. After a series of washes with a gradient of SSC and SDS, the membrane was exposed to a film and developed using a PhosphoImager (Fuji).

### Generation of aP2-B1/B1−/− Mice

Mice with adipose tissue overexpression of kinin B_1_R were generated by pro-nuclear injection of a construct in which the expression of the kinin B_1_R gene was driven by the promoter/enhancer element of the aP2 gene [36] (Figure S1A). Transgenic mice were backcrossed to B_1_^−/−^ mice to generate aP2-B_1_/B_1_^−/−^ lines.

### Microarray Analysis

Microarray analysis was performed as previously described [37]. RNA samples from four independent mice per group were used for cRNA synthesis using Affymetrix cRNA Amplification Kit. cRNA was hybridized onto Affymetrix Genechips M430 2.0. Gene Set Enrichment Analysis (GSEA) was performed using GSEA software (Broad Institute, Cambridge, MA).

### Diets, Metabolic Tests, and Lipid Profiling

Twelve-week-old mice were fed with standard diet (10% kCal fat) or high-fat diet (HFD) (45% kCal fat) (Research Diets). After the treatment, mice were fasted for 12 hours and sacrificed for collection of blood and tissue samples. To perform glucose and insulin tolerance tests mice were fasted overnight and injected intraperitoneally with either 1g glucose or 0.5U insulin (Humalog) per kg body weight. Blood glucose levels were measured using the Accu-Chek Advantage glucometer (Roche). Serum lipid profiles were determined using colorimetric methods (Labtest). Body temperature was measured using a rectal thermometer (YSI mod. 4000). Spontaneous activity signals of freely moving mice were computed at 10-s intervals using a computer-assisted data acquisition program (ADDENFI metabolic chamber prototype). Body composition analysis was performed by dual-energy X-ray absorptiometry using a Hologic QDR 4500 scanner [17].

### Statistical Analysis

Values are mean ± SEM. Statistical analyses were performed using the two-tailed Student's unpaired t test to compare two independent groups, or ANOVA followed by Bonferroni's test to compare more than two. Significance was considered at P<0.05.

## Supporting Information

Figure S1 **Kinin B1R mRNA expression in tissues of high fat diet treated mice.** C57BL/6 mice were submitted to HFD for 9 weeks (n = 3–4 per group). Kinin B1R expression was quantified in the tissues by real time PCR. Results are mean ± SEM. *, P<0.05. Gastrocnemius muscle was studied.
(TIF)

Figure S2 **Generation and characterization of aP2-B1/B1−/− mice.** A: pBSaP2B1pA plasmid harboring the aP2-B1 transgene. The 5.6 kb aP2 promoter/enhancer element (aP2 prom) was cloned upstream of the kinin B1 receptor coding region (B1) followed by the SV40 poly-adenylation site (pA). B: Tail DNA PCR genotyping fragments displaying two transgenic colony founders (25 and 18). pBSaP2B1pA was used as a positive control. C: The genomic DNA of the two founders was isolated and southern blotting was performed to confirm the insertion of the transgene into the genome. pBSaP2B1pA was used as a positive control. D: RNAse Protection Assay was performed to assess the expression of the aP2-B1 transgene in different organs. L32 was used as the loading control. E: Des-Arg9-bradykinin (DBK) binding assays performed in isolated adipocytes. Values are means ± SEM of six animals per group. F: Effects of acute treatment with DBK, the B2 receptor agonist bradykinin (BK) or DBK in the presence of the B1 receptor antagonist [Leu8]-DBK on the extracellular acidification rate of isolated adipocytes from wild type (WT), B1 knockout (B1−/−) and aP2-B1/B1−/− mice (n = 5 animals per group) measured by the Cytosensor system.
(TIF)

Figure S3 **Expression of kinin B_1_ receptor in fat substantially affects adipose tissue gene expression profile.** A: Heat map displaying gene expression in adipose tissue of wild type (WT), B_1_^−/−^ (KO) and aP2-B_1_/B_1_^−/−^ (KO_aP2-B) as assessed by Affymetrix microarrays and analyzed by the Gene Set Enrichment Analysis tool. B: Heat map displaying gene expression of members of the insulin signaling pathway (as annotated in the Kyoto Encyclopedia of Genes and Genomes) in adipose tissue of wild type (WT), B_1_^−/−^ (KO) and aP2-B_1_/B_1_^−/−^ (KO_aP2-B). C: Kinin B_2_ receptor mRNA expression in the adipose tissue based on Affymetrix microarray analysis. Red, upregulated; Blue, downregulated vs. average of samples.
(TIF)

Figure S4 **Lipid and glucose metabolism genes are not rescued by expression of kinin B_1_ receptor in fat.** Expression of GLUT1, fatty acid synthase (FAS), acetyl-CoA carboxylase (ACC) and glycerol-3-phosphate acyltransferase-like (GPAT) mRNA in epididymal fat of random fed mice measured by realtime PCR. Data represent mean ± SEM of six animals per group. *, P<0.05; **, P<0.001.
(TIF)

Figure S5 **aP2-B1/B1−/− mice have smaller adipocytes.** Animals were submitted to HFD and adipocyte size was estimated in histological sections of the epididymal fat pad. Values are means ± SEM of six animals.
(TIF)

Table S1 Gene Set Enrichment Analysis. Gene sets downregulated in B1−/− mice adipose tissue versus WT and aP2B1-B1−/− adipose tissue.
(XLSX)

Table S2 Energy parameters.
(XLSX)

Table S3 Lipid parameters in the blood.
(XLSX)

Supplementary Methods S1 (DOC)

## References

[1] Arner P (2003) The adipocyte in insulin resistance: key molecules and the impact of the thiazolidinediones. Trends Endocrinol Metab 14: 137–145. 12670740 10.1016/s1043-2760(03)00024-9

[2] Tilg H, Moschen AR (2006) Adipocytokines: mediators linking adipose tissue, inflammation and immunity. Nat Rev Immunol 6: 772–783. 16998510 10.1038/nri1937

[3] Waki H, Tontonoz P (2007) Endocrine functions of adipose tissue. Annu Rev Pathol 2: 31–56. 18039092 10.1146/annurev.pathol.2.010506.091859

[4] Yamauchi T, Kamon J, Waki H, Terauchi Y, Kubota N, et al (2001) The fat-derived hormone adiponectin reverses insulin resistance associated with both lipoatrophy and obesity. Nat Med 7: 941–946. 11479627 10.1038/90984

[5] Yang WS, Lee WJ, Funahashi T, Tanaka S, Matsuzawa Y, et al (2001) Weight reduction increases plasma levels of an adipose-derived anti-inflammatory protein, adiponectin. J Clin Endocrinol Metab 86: 3815–3819. 11502817 10.1210/jcem.86.8.7741

[6] Leeb-Lundberg LM, Marceau F, Muller-Esterl W, Pettibone DJ, Zuraw BL (2005) International union of pharmacology. XLV. Classification of the kinin receptor family: from molecular mechanisms to pathophysiological consequences. Pharmacol Rev 57: 27–77. 15734727 10.1124/pr.57.1.2

[7] Bhoola KD, Figueroa CD, Worthy K (1992) Bioregulation of kinins: kallikreins, kininogens, and kininases. Pharmacol Rev 44: 1–80. 1313585

[8] Calixto JB, Medeiros R, Fernandes ES, Ferreira J, Cabrini DA, et al (2004) Kinin B1 receptors: key G-protein-coupled receptors and their role in inflammatory and painful processes. Br J Pharmacol 143: 803–818. 15520046 10.1038/sj.bjp.0706012 PMC1575942

[9] Dietze G, Wicklmayr M (1977) [Effect of bradykinin on muscular glucose uptake in man (author's transl)]. Klin Wochenschr 55: 357–358. 870748 10.1007/BF01488118

[10] Dietze GJ, Wicklmayr M, Rett K, Jacob S, Henriksen EJ (1996) Potential role of bradykinin in forearm muscle metabolism in humans. Diabetes 45 Suppl 1S110–114. 8529790 10.2337/diab.45.1.s110

[11] Miyata T, Taguchi T, Uehara M, Isami S, Kishikawa H, et al (1998) Bradykinin potentiates insulin-stimulated glucose uptake and enhances insulin signal through the bradykinin B2 receptor in dog skeletal muscle and rat L6 myoblasts. Eur J Endocrinol 138: 344–352. 9539311 10.1530/eje.0.1380344

[12] Isami S, Kishikawa H, Araki E, Uehara M, Kaneko K, et al (1996) Bradykinin enhances GLUT4 translocation through the increase of insulin receptor tyrosine kinase in primary adipocytes: evidence that bradykinin stimulates the insulin signalling pathway. Diabetologia 39: 412–420. 8777990 10.1007/BF00400672

[13] Beard KM, Lu H, Ho K, Fantus IG (2006) Bradykinin augments insulin-stimulated glucose transport in rat adipocytes via endothelial nitric oxide synthase-mediated inhibition of Jun NH2-terminal kinase. Diabetes 55: 2678–2687. 17003331 10.2337/db05-1538

[14] Duka I, Shenouda S, Johns C, Kintsurashvili E, Gavras I, et al (2001) Role of the B(2) receptor of bradykinin in insulin sensitivity. Hypertension 38: 1355–1360. 11751717 10.1161/hy1201.096574

[15] Zuccollo A, Navarro M, Frontera M, Cueva F, Carattino M, et al (1999) The involvement of kallikrein-kinin system in diabetes type I (insulitis). Immunopharmacology 45: 69–74. 10614992 10.1016/s0162-3109(99)00149-6

[16] Araujo RC, Mori MA, Merino VF, Bascands JL, Schanstra JP, et al (2006) Role of the kinin B1 receptor in insulin homeostasis and pancreatic islet function. Biol Chem 387: 431–436. 16606341 10.1515/BC.2006.057

[17] Mori MA, Araujo RC, Reis FC, Sgai DG, Fonseca RG, et al (2008) Kinin B1 receptor deficiency leads to leptin hypersensitivity and resistance to obesity. Diabetes 57: 1491–1500. 18332096 10.2337/db07-1508

[18] Dias JP, Couture R (2012) Blockade of kinin B(1) receptor reverses plasma fatty acids composition changes and body and tissue fat gain in a rat model of insulin resistance. Diabetes Obes Metab 14: 244–253. 22023455 10.1111/j.1463-1326.2011.01521.x

[19] Cassis LA, Police SB, Yiannikouris F, Thatcher SE (2008) Local adipose tissue renin-angiotensin system. Curr Hypertens Rep 10: 93–98. 18474174 10.1007/s11906-008-0019-9 PMC2702858

[20] Kishi K, Muromoto N, Nakaya Y, Miyata I, Hagi A, et al (1998) Bradykinin directly triggers GLUT4 translocation via an insulin-independent pathway. Diabetes 47: 550–558. 9568686 10.2337/diabetes.47.4.550

[21] Subramanian A, Tamayo P, Mootha VK, Mukherjee S, Ebert BL, et al (2005) Gene set enrichment analysis: a knowledge-based approach for interpreting genome-wide expression profiles. Proc Natl Acad Sci U S A 102: 15545–15550. 16199517 10.1073/pnas.0506580102 PMC1239896

[22] Duka A, Kintsurashvili E, Duka I, Ona D, Hopkins TA, et al (2008) Angiotensin-converting enzyme inhibition after experimental myocardial infarct: role of the kinin B1 and B2 receptors. Hypertension 51: 1352–1357. 18347228 10.1161/HYPERTENSIONAHA.107.108506

[23] Merino VF, Todiras M, Mori MA, Sales VM, Fonseca RG, et al (2009) Predisposition to atherosclerosis and aortic aneurysms in mice deficient in kinin B1 receptor and apolipoprotein E. J Mol Med (Berl). 87: 953–963. 10.1007/s00109-009-0501-0 19618151

[24] Prada PO, Hirabara SM, de Souza CT, Schenka AA, Zecchin HG, et al (2007) L-glutamine supplementation induces insulin resistance in adipose tissue and improves insulin signalling in liver and muscle of rats with diet-induced obesity. Diabetologia 50: 1949–1959. 17604977 10.1007/s00125-007-0723-z

[25] Shi H, Cave B, Inouye K, Bjorbaek C, Flier JS (2006) Overexpression of suppressor of cytokine signaling 3 in adipose tissue causes local but not systemic insulin resistance. Diabetes 55: 699–707. 16505233 10.2337/diabetes.55.03.06.db05-0841

[26] Xu H, Hirosumi J, Uysal KT, Guler AD, Hotamisligil GS (2002) Exclusive action of transmembrane TNF alpha in adipose tissue leads to reduced adipose mass and local but not systemic insulin resistance. Endocrinology 143: 1502–1511. 11897709 10.1210/endo.143.4.8715

[27] Bluher M, Michael MD, Peroni OD, Ueki K, Carter N, et al (2002) Adipose tissue selective insulin receptor knockout protects against obesity and obesity-related glucose intolerance. Dev Cell 3: 25–38. 12110165 10.1016/s1534-5807(02)00199-5

[28] Trayhurn P, Beattie JH (2001) Physiological role of adipose tissue: white adipose tissue as an endocrine and secretory organ. Proc Nutr Soc 60: 329–339. 11681807 10.1079/pns200194

[29] Pesquero JB, Araujo RC, Heppenstall PA, Stucky CL, Silva JA Jr, et al (2000) Hypoalgesia and altered inflammatory responses in mice lacking kinin B1 receptors. Proc Natl Acad Sci U S A 97: 8140–8145. 10859349 10.1073/pnas.120035997 PMC16683

[30] Rodbell M (1964) Metabolism of Isolated Fat Cells. I. Effects of Hormones on Glucose Metabolism and Lipolysis. J Biol Chem 239: 375–380. 14169133

[31] McConnell HM, Owicki JC, Parce JW, Miller DL, Baxter GT, et al (1992) The cytosensor microphysiometer: biological applications of silicon technology. Science 257: 1906–1912. 1329199 10.1126/science.1329199

[32] Lima FB, Bao S, Garvey WT (1994) Biological actions of insulin are differentially regulated by glucose and insulin in primary cultured adipocytes. Chronic ability to increase glycogen synthase activity. Diabetes 43: 53–62. 8262317 10.2337/diab.43.1.53

[33] Alonso-Vale MI, Andreotti S, Mukai PY, Borges-Silva CN, Peres SB, et al (2008) Melatonin and the circadian entrainment of metabolic and hormonal activities in primary isolated adipocytes. J Pineal Res 45: 422–429. 18662218 10.1111/j.1600-079X.2008.00610.x

[34] DeBlasi A, O'Reilly K, Motulsky HJ (1989) Calculating receptor number from binding experiments using same compound as radioligand and competitor. Trends Pharmacol Sci 10: 227–229. 2773043 10.1016/0165-6147(89)90266-6

[35] Bogan JS, McKee AE, Lodish HF (2001) Insulin-responsive compartments containing GLUT4 in 3T3-L1 and CHO cells: regulation by amino acid concentrations. Mol Cell Biol 21: 4785–4806. 11416153 10.1128/MCB.21.14.4785-4806.2001 PMC87167

[36] Ross SR, Graves RA, Greenstein A, Platt KA, Shyu HL, et al (1990) A fat-specific enhancer is the primary determinant of gene expression for adipocyte P2 in vivo. Proc Natl Acad Sci U S A 87: 9590–9594. 2263614 10.1073/pnas.87.24.9590 PMC55218

[37] Mori MA, Liu M, Bezy O, Almind K, Shapiro H, et al (2010) A systems biology approach identifies inflammatory abnormalities between mouse strains prior to development of metabolic disease. Diabetes 59: 2960–2971. 20713682 10.2337/db10-0367 PMC2963557

