# Supplementary figures and images for: Kinin B1 Receptor in Adipocytes Regulates Glucose Tolerance and Predisposition to Obesity
Source: PLoS One. 2012 Sep 14;7(9):e44782. doi: 10.1371/journal.pone.0044782 (PMC3443087; doi:10.1371/journal.pone.0044782)

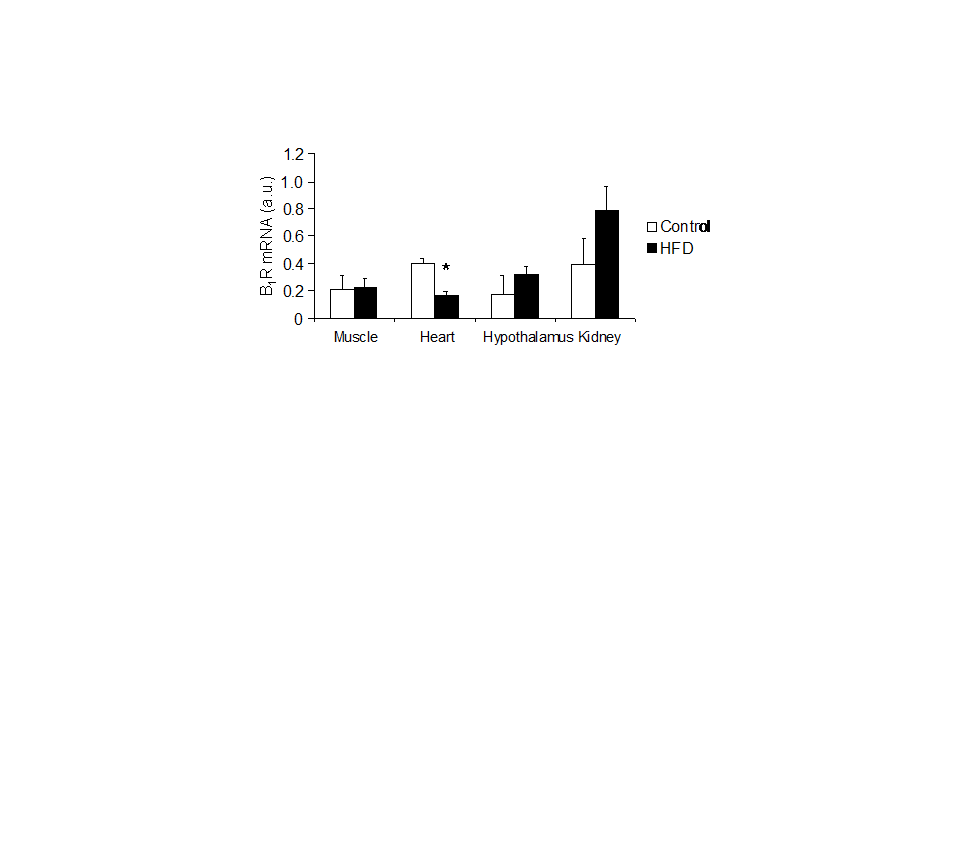

Supplement: Figure S1 — Kinin B1R mRNA expression in tissues of high fat diet treated mice. C57BL/6 mice were submitted to HFD for 9 weeks (n = 3–4 per group). Kinin B1R expression was quantified in the tissues by real time PCR. Results are mean ± SEM. *, P<0.05. Gastrocnemius muscle was studied. (TIF) [file pone.0044782.s001.tif]

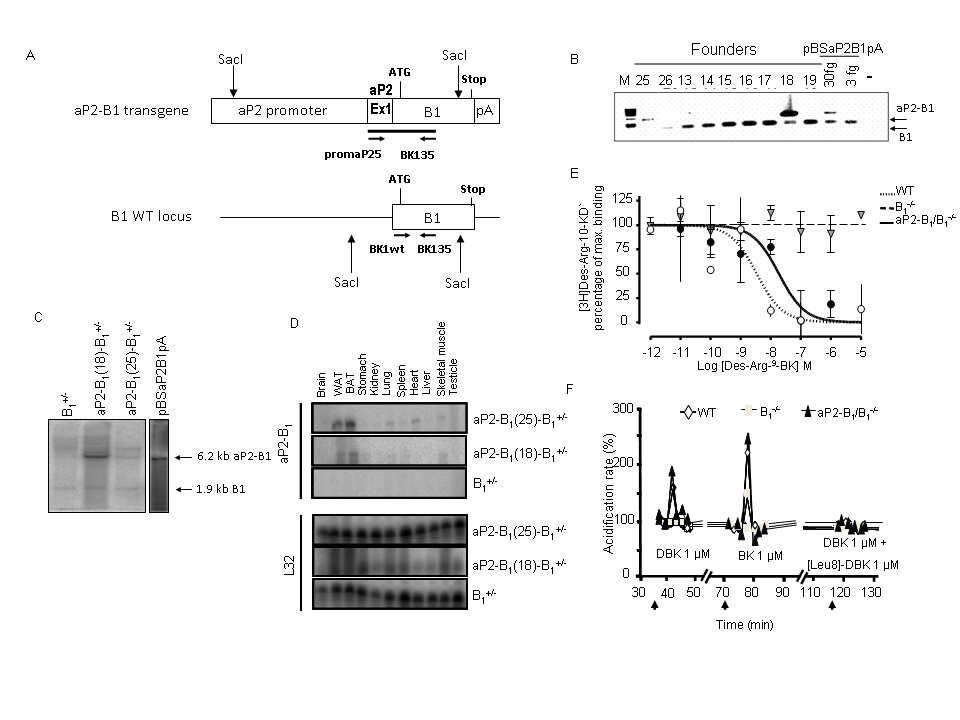

Supplement: Figure S2 — Generation and characterization of aP2-B1/B1−/− mice. A: pBSaP2B1pA plasmid harboring the aP2-B1 transgene. The 5.6 kb aP2 promoter/enhancer element (aP2 prom) was cloned upstream of the kinin B1 receptor coding region (B1) followed by the SV40 poly-adenylation site (pA). B: Tail DNA PCR genotyping fragments displaying two transgenic colony founders (25 and 18). pBSaP2B1pA was used as a positive control. C: The genomic DNA of the two founders was isolated and southern blotting was performed to confirm the insertion of the transgene into the genome. pBSaP2B1pA was used as a positive control. D: RNAse Protection Assay was performed to assess the expression of the aP2-B1 transgene in different organs. L32 was used as the loading control. E: Des-Arg9-bradykinin (DBK) binding assays performed in isolated adipocytes. Values are means ± SEM of six animals per group. F: Effects of acute treatment with DBK, the B2 receptor agonist bradykinin (BK) or DBK in the presence of the B1 receptor antagonist [Leu8]-DBK on the extracellular acidification rate of isolated adipocytes from wild type (WT), B1 knockout (B1−/−) and aP2-B1/B1−/− mice (n = 5 animals per group) measured by the Cytosensor system. (TIF) [file pone.0044782.s002.tif]

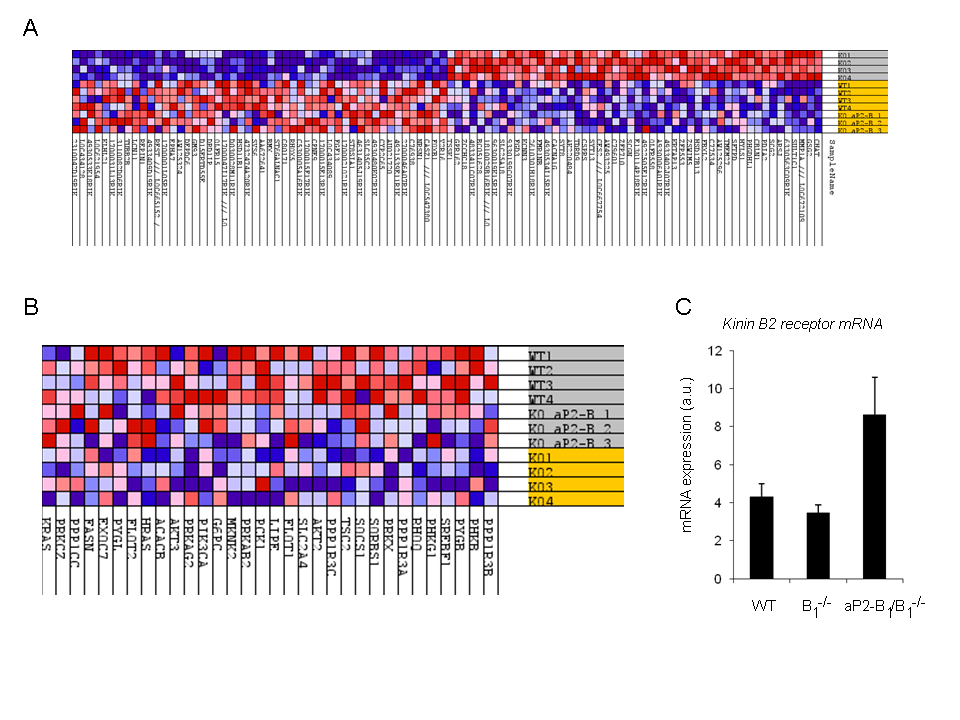

Supplement: Figure S3 — Expression of kinin B1 receptor in fat substantially affects adipose tissue gene expression profile. A: Heat map displaying gene expression in adipose tissue of wild type (WT), B1 −/− (KO) and aP2-B1/B1 −/− (KO_aP2-B) as assessed by Affymetrix microarrays and analyzed by the Gene Set Enrichment Analysis tool. B: Heat map displaying gene expression of members of the insulin signaling pathway (as annotated in the Kyoto Encyclopedia of Genes and Genomes) in adipose tissue of wild type (WT), B1 −/− (KO) and aP2-B1/B1 −/− (KO_aP2-B). C: Kinin B2 receptor mRNA expression in the adipose tissue based on Affymetrix microarray analysis. Red, upregulated; Blue, downregulated vs. average of samples. (TIF) [file pone.0044782.s003.tif]

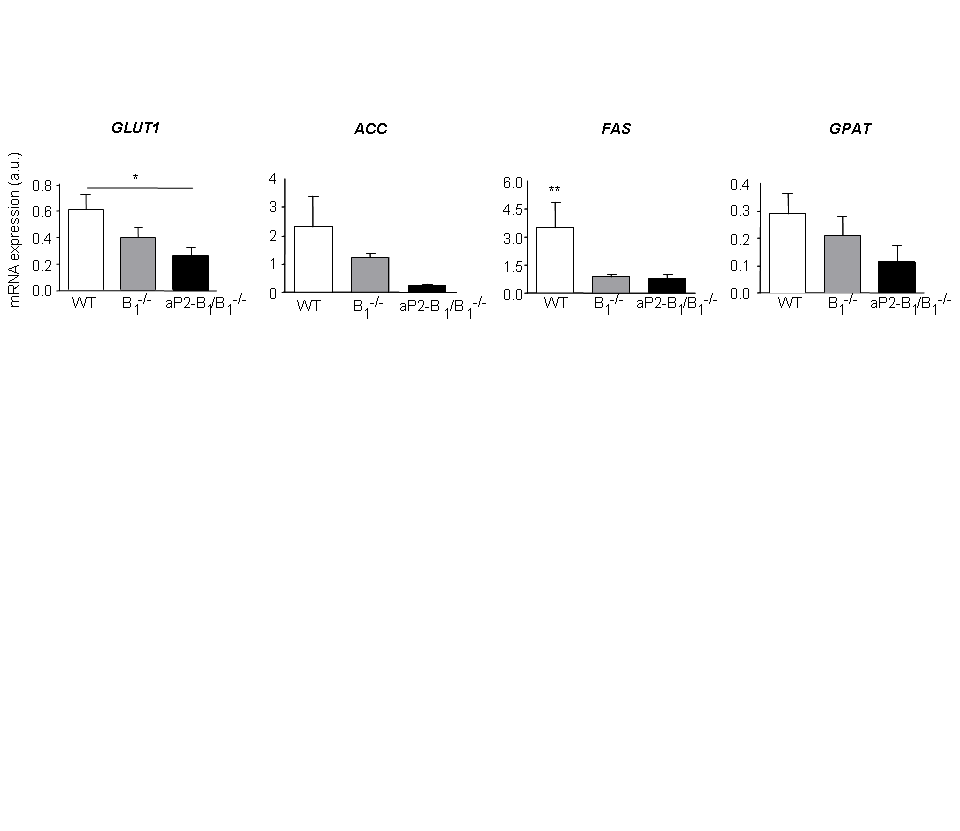

Supplement: Figure S4 — Lipid and glucose metabolism genes are not rescued by expression of kinin B1 receptor in fat. Expression of GLUT1, fatty acid synthase (FAS), acetyl-CoA carboxylase (ACC) and glycerol-3-phosphate acyltransferase-like (GPAT) mRNA in epididymal fat of random fed mice measured by realtime PCR. Data represent mean ± SEM of six animals per group. *, P<0.05; **, P<0.001. (TIF) [file pone.0044782.s004.tif]

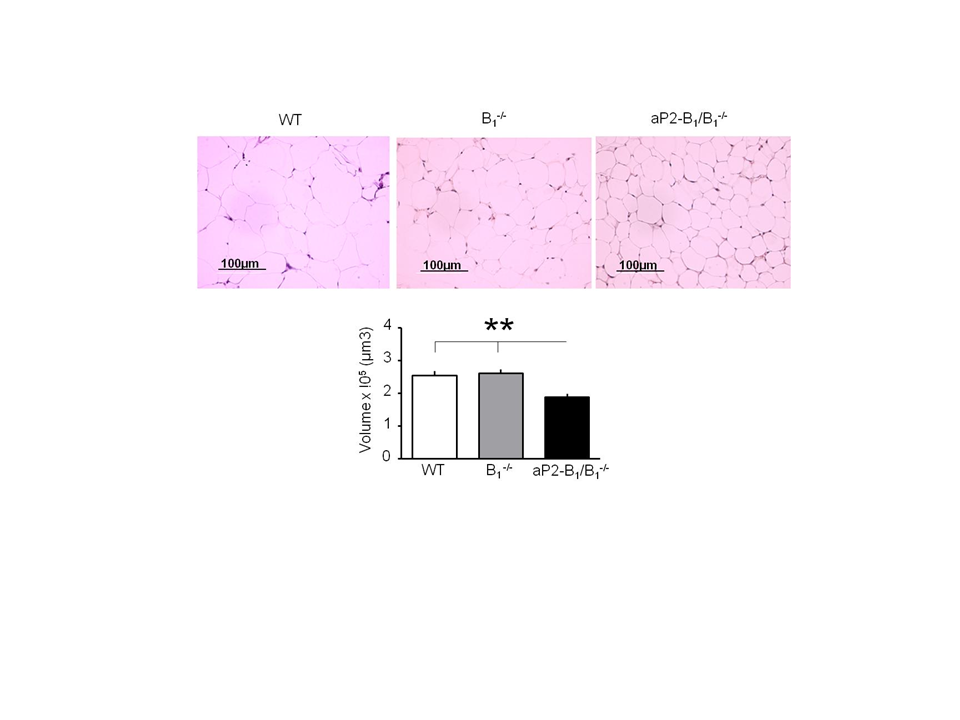

Supplement: Figure S5 — aP2-B1/B1−/− mice have smaller adipocytes. Animals were submitted to HFD and adipocyte size was estimated in histological sections of the epididymal fat pad. Values are means ± SEM of six animals. (TIF) [file pone.0044782.s005.tif]
