# Supplementary material for: Kinin B1 Receptor in Adipocytes Regulates Glucose Tolerance and Predisposition to Obesity
Source: PLoS One. 2012 Sep 14;7(9):e44782. doi: 10.1371/journal.pone.0044782 (PMC3443087; doi:10.1371/journal.pone.0044782)
Supplement: Supplementary Methods S1 — (DOC) [file pone.0044782.s009.doc]

**Supplementary methods**

**RT-PCR**

Total RNA was isolated using TRizol reagent (Invitrogen) and treated with DNAse I (Invitrogen) according to the manufacturer’s protocol. Reverse transcription was performed using 1 μg of total RNA, 200 U M-MLV reverse transcriptase (Invitrogen), 5 mM dithiothreitol, 50 ng random hexamers primers, 1x First Strand buffer, 0.5 mM dNTP, and 3 mM MgCl_2_ (reagents provided with the enzyme). Reactions were subjected to the following protocol: 20 ºC/ 10 min, 42 ºC/ 45 min, 95 ºC/ 5 min and 4 ºC/ 10 min. Resultant cDNA was then used for PCRs as described below. TaqMan PCR for quantitative analysis of mouse kinin B_1_ receptor and β-actin mRNA expression, as well as SYBR-Green quantitative PCRs were performed as described elsewhere [1]. Standard semi-quantitative PCRs were performed to detect mRNA expression of the components of the kallikrein-kinin system in WAT of mice. Sequences of the primers used were: β-actin (mouse: 5’-CGAGGCCCAGAGCAAGAGAG-3’; 5’-AGGAAGAGGATGCGGCAGTGG-3’), kallikrein (5’-CTACAACATGAGCCTCCATCG-3’; 5’-CAGACAGTGTGTCACTCAGG –3’), carboxypeptidase M (5’- AGCCCGACTGTTACTACAGC-3’; 5’-TCAGCGCGCACTTTTATATATTCGAT-3’), B2 receptor (5’-ATCCTCACTCCTCTTTGTCC-3’; 5’-GGTCCTGAACACCAACATGG-3’); 36B4 (5’-AACCCTGAAGTGCTCGACAT-3’, 5’-CCGATCTGCAGACACACACT-3’); Acrp30 (5’-CGCGTCACTGTTCCCAATGTA-3’, 5’-CCAGTGCTGCCGTCATAATGA-3’); GLUT-4 (5′-TCTACATCATCCGGAACCTGGA-3′, 5′-TCAGGCGCTTTAGACTCTTTGG-3′); GLUT-1 (5’-CTATTGCTGTGGCTGGCTTC-3’, 5’-GAAGAAGAGCACGAGGAGCA-3’); ACC (5’-ATCCGCCTCTTCCTGACAA-3’, 5’-TGCCTGGAACCTCTTTGATT-3’); FAS (5’-GCAAGCTGTCCCCTGATG-3’, 5’-GAACCAGCCCCATCACAC-3’); GPAT (5’-CCCTGCCAGACTTTTTACCA-3’, 5’-GCTTCTTGTCCCACTGCTG-3’). PCRs were set using 0.2 μg of total cDNA, 0.3 U Taq DNA polymerase (Invitrogen), 1x PCR buffer, 0.5 mM dNTPs, and 2.5 mM MgCl_2_ (reagents provided with the enzyme) and 50 ng primers. Reactions were subjected to 95 ºC/ 5 min and 35 cycles of 95 ºC/ 20 s, 60 ºC/ 20 s and 72 ºC/ 40 s.

**Antibodies**

AKT (#9272), phospho-AKT (Ser473) (#9271), ERK (#9102) and phospho-ERK (Thr202, Tyr204) (#9101) antibodies were purchased from Cell Signaling Technologies. GLUT4 (#AB1346) antibody was obtained from Chemicon. IRS-1 (#06-248) and p85 PI3K subunit (#06-195) antibodies were purchased from Upstate.

**Isolation of mouse adipocytes**

Adipocytes were isolated from epididymal fat pads using a slight modification of Rodbell’s method [2]. Briefly, mice were killed and epididymal fat pads were removed, minced and incubated for 15 minutes at 37 ºC under orbital shaking (150 rpm) in DMEM with 5 mM glucose containing 1.25 mg/mL collagenase, 25 mM HEPES and 4 % BSA (Sigma), pH 7.4. At the end of the incubation, cells were filtered through a 500 μm nylon mesh, washed three times with DMEM with 5 mM glucose containing 20 mM HEPES, 1 % BSA and 10 % FBS, pH 7.4, and the resultant suspension containing the fat cells was used for experiments. Images of isolated adipocytes were acquired using a light microscope fitted with a camera and the cellular volume was estimated using a graduated ocular according to the DiGirolamo’s method [3].

**Protein extraction, immunoprecipitation and western blotting**

Tissues and cells were homogenized at 4 °C in RIPA buffer [50 mM tris, pH 7.4; 1 % NP-40; 0.25 % sodium deoxycholate; 150 mM NaCl; 1 mM EDTA; Complete protease inhibitors (Roche); 1 mM sodium orthovanadate; 1 mM sodium fluoride] and protein content in the lysates was quantified using the Bio-Rad DC Protein Assay kit. Cell lysates were diluted in RIPA buffer and 4 x loading buffer (125 mM tris pH 6.8; 1.25 % glycerol; 4 % sodium dodecyl sulfate; 15 mg/mL dithiothreitol; 0.02 % bromophenol blue) to a final concentration of 5 mg/mL and boiled. 10 μL of the lysates were subjected to SDS-PAGE, transferred to PVDF membranes (GE Healthcare) and immunoblotted. Horseradish peroxidase activity was detected by chemiluminescence using the ECL Plus kit. Alternatively, 500 μL of lysates containing 1 mg of total protein were incubated overnight at 4°C with 25 μL of Protein G-sepharose beads (GE Healthcare) and 4 μg of anti-PI3K antibody (Upstate). In the following day, samples were washed four times with cold RIPA and the pellet was resuspended in 2 x loading buffer and boiled. The supernatant was analyzed by SDS-PAGE as described above. Low density microsomes and plasma membrane fractions from 3T3-L1 adipocytes and WAT of wild type (WT) and B1-/- mice were isolated for quantification of GLUT4 expression based on a protocol described elsewhere [4]. Total protein content was determined in the solubilized membrane fractions and equal amounts of protein were analyzed by SDS-PAGE and immunoblotting.

**RNase protection assay**

Expression of the aP2-B1 transgene was determined using RNase protection assay (RPA III kit, Ambion). The probe corresponded to the antisense transcript resulted of the amplification of the transgene using the following primers: 5’-TGGACTTCAGAGGCTCATAG-3’ and 5’-CCAGCAACCTGTAGCGGTCC-3’. The PCR fragment was cloned into pGEM-T Easy vector and used as template for in vitro transcription. Briefly, the probe was labeled with α-[32P]-UTP and incubated overnight at 42 °C with 50 μg of total RNA. The incubation products were subjected to 5% acrylamide / 8M urea gel electrophoresis. The gel was dried out, exposed to an X-ray film for 16 hours and developed using a PhosphoImager (Fuji).

**Southern Blotting**

The probe was synthesized by PCR using an aP2-B1 transgene-specific set of primers (5’-TGGACTTCAGAGGCTCATAG-3’ and 5’-CCAGCAACCTGTAGCGGTCC-3’). The fragment was purified through a silica gel column and labeled with α-[32P]-CTP using the Prime-It kit Random Primer Labeling RmT (Ambion). The genomic DNA was extracted using DNeasy kit (Qiagen), digested with Sac I and subjected to 1% agarose gel electrophoresis. The gel was dipped in a solution of 0.2 M HCl for 10 minutes for depurination of the DNA, rinsed briefly with water, incubated for 30 min with denaturation solution (1.5 M NaCl, 0.5 M NaOH) and then for another 30 minutes with neutralizing solution (1.5 M NaCl, 1 M Tris, pH 7.5). DNA was transferred by capillarity from the gel to a Hybond-N nylon membrane (GE Healthcare) and cross-linked using ultraviolet light. The membrane was incubated overnight at 62°C with an incubation solution (50 mM Tris pH 8.0, 10 mM EDTA, 5 x SSC, 1 x Denhardt’s, 0.2 % SDS, 100 mg/mL salmon sperm DNA) containing the labeled probe. After a series of washes with a gradient of SSC and SDS (2 x SSC 0.5% SDS, 0.5 x SSC 0.1% SDS, 0.1 x SSC 0.1% SDS), the membrane was exposed to a film for 16 hours and developed using a PhosphoImager (Fuji).

**Histology**

Adipose tissues were extracted, weighed and fixed with 10% buffered formalin and embedded in paraffin. Sections were stained with hematoxylin and eosin. Image capture and analysis were performed using ImageJ software.

Supplemental references

1. Mori MA, Araujo RC, Reis FC, Sgai DG, Fonseca RG, et al. (2008) Kinin B1 receptor deficiency leads to leptin hypersensitivity and resistance to obesity. Diabetes 57: 1491-1500.

2. Rodbell M (1964) Metabolism of Isolated Fat Cells. I. Effects of Hormones on Glucose Metabolism and Lipolysis. J Biol Chem 239: 375-380.

3. Fine JB, DiGirolamo M (1997) A simple method to predict cellular density in adipocyte metabolic incubations. Int J Obes Relat Metab Disord 21: 764-768.

4. Bogan JS, McKee AE, Lodish HF (2001) Insulin-responsive compartments containing GLUT4 in 3T3-L1 and CHO cells: regulation by amino acid concentrations. Mol Cell Biol 21: 4785-4806.
